# TLR7 Expression Is Associated with M2 Macrophage Subset in Calcific Aortic Valve Stenosis

**DOI:** 10.3390/cells9071710

**Published:** 2020-07-16

**Authors:** Glykeria Karadimou, Oscar Plunde, Sven-Christian Pawelzik, Miguel Carracedo, Per Eriksson, Anders Franco-Cereceda, Gabrielle Paulsson-Berne, Magnus Bäck

**Affiliations:** 1 Laboratory of Immunobiology, Cardiovascular Medicine Unit, Department of Medicine, Solna, Karolinska Institutet and Karolinska University Hospital, Bioclinicum J8:20, Visionsgatan 4, 171 64 Stockholm, Sweden; glykeria.karadimou@ki.se; 2 Cardiovascular Medicine Unit, Center for Molecular Medicine, Department of Medicine, Solna, Karolinska Institutet and Karolinska University Hospital, 171 77 Stockholm, Sweden; oscar.persson@ki.se (O.P.); sven-christian.pawelzik@ki.se (S.-C.P.); miguel.carracedoortiz@gmail.com (M.C.); per.eriksson@ki.se (P.E.); magnus.back@ki.se (M.B.); 3 Theme Heart and Vessels, Division of Valvular and Coronary Disease, Karolinska University Hospital, 171 77 Stockholm, Sweden; 4 Cardiothoracic Surgery Unit, Department of Molecular Medicine and Surgery, Karolinska Institutet & Karolinska University Hospital, 171 77 Stockholm, Sweden; Anders.Franco-cereceda@ki.se

**Keywords:** macrophage, calcific aortic valve stenosis, cytokine, Toll-like Receptor 7, inflammation

## Abstract

Calcific aortic valve stenosis (CAVS) is a common age-related disease characterized by active calcification of the leaflets of the aortic valve. How innate immune cells are involved in disease pathogenesis is not clear. In this study we investigate the role of the pattern recognition receptor Toll-like receptor 7 (TLR7) in CAVS, especially in relation to macrophage subtype. Human aortic valves were used for mRNA expression analysis, immunofluorescence staining, or ex vivo tissue assays. Response to TLR7 agonist in primary macrophages and valvular interstitial cells (VICs) were investigated in vitro. In the aortic valve, TLR7 correlated with M2 macrophage markers on mRNA levels. Expression was higher in the calcified part compared with the intermediate and healthy parts. TLR7^+^ cells were co-stained with M2-type macrophage receptors CD163 and CD206. Ex vivo stimulation of valve tissue with the TLR7 ligand imiquimod significantly increased secretion of IL-10, TNF-α, and GM-CSF. Primary macrophages responded to imiquimod with increased secretion of IL-10 while isolated VICs did not respond. In summary, in human aortic valves TLR7 expression is associated with M2 macrophages markers. Ex vivo tissue challenge with TLR7 ligand led to secretion of immunomodulatory cytokine IL-10. These results connect TLR7 activation in CAVS to reduced inflammation and improved clearance.

## 1. Introduction

Calcific aortic valve stenosis (CAVS) is classified third in prevalence among cardiovascular diseases [1]. The most important risk factor for developing CAVS is age, although other factors such as genetics, hypertension, sex, smoking, and renal dysfunction also play an important role in the pathophysiology of the disease [2,3,4,5,6]. CAVS is characterized by initial thickening of the aortic valve leaflets because of extracellular matrix remodeling and in progressed stages by calcific depositions [7]. Valvular interstitial cells (VICs), the most abundant cell type in the aortic valve, actively contribute to calcification [2,8]. Currently, there is no medical treatment; instead, patients rely on invasive replacement of the aortic valve. However, replacement of the valve cannot fully reverse heart remodeling [2,9,10] and the lack of medical treatments demand large health care recourses.

Stenotic aortic valves share several morphological characteristics with atherosclerotic lesions, including a strong inflammatory component. Inflammatory cells such as T cells, B cells, and macrophages and their respective pro- or anti-inflammatory mediators are present in diseased aortic valves. The role of immune cells in CAVS is not fully understood. Infiltration of T and B cells takes place in advanced stages of aortic valve stenosis and has been associated with the promotion of pro-inflammatory processes [11]. Macrophages are the immune cell type that is mainly infiltrating aortic valves in large numbers [2,12]. They were initially thought to be associated with detrimental effects in cardiovascular disease; however, data from several areas show that macrophages are also necessary players in healing and in inflammation resolution in the aortic valve [13,14]. Macrophages respond fast through pattern recognition receptors such as the Toll-like receptors (TLRs), a gene family with 10-14 members [15]. Furthermore, T and B cells express several TLRs [16].

The effect that activation of a TLR member has on CAVS is not fully understood. In vitro studies have shown that VICs can respond to ligands for TLR2 and TLR4, respectively, by NF-κB activation and increased secretion of pro-osteogenic factors such as IL-6 and ICAM-1 [17]. While both TLR2 and TLR4 are cell surface receptors, TLR7 belongs to the group of intracellular Toll-like receptors, located in the endolysosome. The ligands that have been identified for TLR7 so far are single stranded RNA and imidazoquinoline derivatives [18]. Interestingly, TLR7 has been shown to be protective in atherosclerosis [19,20,21]. In human carotid atherosclerotic lesions TLR7 was correlated with M2 macrophage subtype [20]. Furthermore, TLR7 mRNA levels in carotid atherosclerotic plaques were associated with fewer major cardio- and cerebrovascular events (MACCE). In the same study, TLR7 was expressed on macrophages and T cells in carotid atherosclerotic plaques [19].

The previously mentioned studies implicate that TLR7 could exert a protective role by association with tissue repair pathways through macrophages. In this study, we aim (1) to determine the expression of TLR7 in human aortic valves affected by CAVS, (2) to identify potential associations of TLR7 with the different immune cell populations, (3) to explore the activation of TLR7 in human calcified aortic valves, and (4) to identify the cell types that respond to TLR7 activation in CAVS.

## 2. Materials and Methods

### 2.1. Reagents

Reagents for aortic valve tissue analysis were purchased from Qiagen (Hilden, Germany): RNA Later (Cat. # 76104) and RNeasy Lipid Tissue Mini kit (Cat. #74804). Cell culture reagents were purchased from Gibco/ThermoScientific (Waltham, MA, USA): phenol red free Dulbecco’s modified Eagle medium (DMEM, Cat. #31053028), RPMI 1640 medium (Cat. #31870025), fetal bovine serum (FBS, Cat. #10270106), penicillin-streptomycin (10,000 U/mL, Cat. #15140122), HEPES (Cat. #15630056), sodium pyruvate (Cat. #11360070), and L-glutamine (Cat. #25030081). Primary antibodies were purchased from Abcam (Cambridge, UK; Anti-CD163 antibody (Cat. #ab74604)), Agilent (Santa Clara, CA, USA; Anti-CD68 (Dako Omnis) (Cat. #M0876)), R&D systems (Minneapolis, MN, USA; Human MR/CD206 Antibody (Cat. #AF2534)), Novus Biologicals (Cambridge, UK; Anti-TLR7 antibody (Cat. #NBP2-24906)), and Biocare Medical (Pacheco, CA, USA; human Anti-CD3 antibody (Cat.#PM110)) respectively. Secondary antibodies were purchased from Vector Laboratories (Burlingame, CA, USA): horse anti-mouse IgG antibody (H + L), DyLight® 488 (Cat. #DI-2488) and goat anti-rabbit IgG antibody (H + L), DyLight® 594 (Cat. #DI-1594). The TLR7 agonist imiquimod (IMQ, Cat. #tlrl-imq) was purchased from Invivogen (Toulouse, France), and the TLR7 inhibitor ODN20958 (Cat. #130-105-820) was purchased from Miltenyi (Bergisch Gladbach, Germany). Human IL-10 DuoSet ELISA Kit (Cat. #DY217B), human TNF-alpha DuoSet ELISA Kit (Cat. #DY210), human GM-CSF DuoSet ELISA Kit (Cat. #DY215), and human IL-8/CXCL8 DuoSet ELISA Kit (Cat. # DY208) as well as recombinant human M-CSF protein, CF (Cat. #216-MC/CF) were purchased from R&D systems (Minneapolis, MN, USA). All other reagents were purchased from SigmaAldrich (St. Louis, MO, USA).

### 2.2. Collection and Macroscopic Dissection of Human Tricuspid Aortic Valves

Human tricuspid aortic valves were obtained after written consent according to the Declaration of Helsinki and the local ethics committee (2012/1633-31/4). Valves from *n* = 55 patients undergoing aortic valve replacement surgery at the Karolinska University Hospital in Stockholm, Sweden, were collected in RNA Later (Qiagen, Hilden, Germany) immediately after surgery and stored at 4 °C. Upon arrival in the laboratory, the valves were dissected into three parts; healthy, intermediate, and calcified as previously described [22,23]. For histological analysis, valves (*n* = 7) were collected in phenol red free Dulbecco’s modified Eagle medium (DMEM, Gibco, ThermoScientific, Waltham, MA, USA), fixed in 4% PBS-buffered formalin, embedded in paraffin, and cut into 5-µm thick sections. For the ex vivo culture assay, fresh aortic valves (*n* = 5) were collected in DMEM and processed upon arrival in the laboratory.

### 2.3. Valve mRNA Expression Microarrays

Total RNA from aortic valves was isolated using the RNeasy lipid tissue mini kit (Qiagen, Hilden, Germany). RNA concentration was measured using a NanoDrop spectrophotometer (ThermoScientific, Waltham, MA, USA), and RNA quality was assessed by a 2100 bioanalyzer (Agilent, Santa Clara, CA, USA). Total of 100 ng total RNA was sent for array analysis. Valve gene expression data were obtained using Gene Chip Affymetrix human transcriptome 2.0 arrays (HTA 2.0, Santa Clara, CA, USA) and normalized with signal space transformation-robust multi-chip analysis (SST-RMA) using Expression Console (Affymetrix, Santa Clara, CA, USA), as previously described [24].

### 2.4. Immunofluorescence

Serial paraffin sections of human aortic valve specimens were deparaffinized and hydrated in decreasing ethanol concentrations (99%, 95%, 70%). Heat-induced antigen retrieval was performed, and sections were blocked with 5% serum for 30 min in RT. Next, sections were incubated overnight at 4 °C with primary antibodies against CD163 (pre-diluted, Abcam, Cambridge, UK), CD68 (1:50, Dako, Glostrup, Denmark), CD3 (pre-diluted, Biocare Medical, Pacheco, CA, USA ), and CD206 (1:50, R&D Systems, Minneapolis, USA) followed by an overnight incubation at 4 °C with TLR7 antibody (1:100, Novus Biologicals, Cambridge, UK). Sections were incubated with the following secondary antibodies: Dylight 488 anti-mouse and Dylight 594 anti-rabbit (1:300) (Vector Laboratories, Burlingame, CA). All antibodies were diluted in Tris buffered saline with Tween 20 supplemented with 5% horse serum. Autofluorescence was blocked with 0.03% Sudan Black, and DAPI (Dako, Glostrup, Denmark) was used for nuclear staining. Sections were analyzed with a Nikon Eclipse Ti2 confocal microscope.

### 2.5. Ex Vivo Human Aortic Valve Stimulation with TLR7 Agonist

For the cytokine release assay: On operation day, the valve was cut into small pieces (~2 mm^3^), distributed into the wells of a 48-well plate, and thereafter pre-incubated for 1 h in RPMI 1640 medium supplemented with 10% FBS at 37 °C in 5% CO_2_ before stimulation with 12.5 µg/mL TLR7 agonist imiquimod (IMQ, Invivogen, Toulouse. France). To block TLR7 activation the minced valve tissue was pre-incubated for 1 h with 5 µM of a TLR7 inhibitor (ODN 20958, Miltenyi, Bergisch Gladbach, Germany) before adding the TLR7 agonist IMQ (12.5 µg/mL). Each stimulation was run in duplicates. After 20 h of stimulation, supernatants from the valves were collected and stored at −80 °C until cytokine analysis. Supernatant from unstimulated tissue was used as control.

### 2.6. Analysis of Cytokine Secretion

IL-10, TNF-α, GM-CSF, and IL-8 were measured using ELISA kits (R&D systems, Minneapolis, MN, USA) according to the manufacturer´s protocol. Optical absorbance at 450 nm was measured by a Victor Spectrophotometer (PerkinElmer, Waltham, MA, USA).

### 2.7. In Vitro Cell Differentiation and Stimulation

VICs were isolated from five human aortic valves as previously described [23]. In short, the tissue was digested for 16 h incubation with an enzymatic cocktail containing collagenase I and dispase II (Sigma, St. Louis, MO, USA). Isolated VICs were transferred to polystyrene tissue culture flasks and cultured in DMEM containing 10% FBS, 100 units/mL penicillin, 100 µg/mL streptomycin, 1 mM sodium pyruvate, 10 mM HEPES, and 2 mM L-glutamine (Gibco, Waltham, MA, USA). Culture medium was changed every 48 h. Cells were used for in vitro assays between passages 1 and 3.

Monocytes were isolated from buffy coats from four healthy donors that were obtained from the Blood Center of Karolinska University Hospital. Peripheral blood mononuclear cells (PBMC) were isolated by gradient centrifugation. PBMC were reconstituted in PBS supplemented with Ca^2+^ and Mg^2+^ and transferred to polystyrene culture plates, which were transferred for 2 h to 37 °C, 5% CO_2_ to facilitate monocyte attachment to the plate. The wells were washed twice with PBS to remove non-adherent cells, and the cells were provided with RPMI supplemented with 5% FBS, 100 units/mL penicillin, 100 µg/mL streptomycin, and 20 ng/mL M-CSF (R&D systems, Minneapolis, MN) for differentiation into macrophages. After each 48 h incubation period, the plates were washed with culture medium and fresh RPMI supplemented with 5% FBS, 100 units/mL penicillin, 100 µg/mL streptomycin, and 20 ng/mL M-CSF was added. On day 6 macrophages were used for in vitro assays.

VICs and primary macrophages were stimulated with 12.5 µg/mL IMQ. For the VIC stimulation, 100 ng/mL LPS was used as positive control. After 24 h stimulation, the supernatant was collected for cytokine measurements.

### 2.8. Statistical Analysis 

Samples were analyzed by the Student´s t-test and 1-way RM ANOVA with Holm-Sidak post-test for comparison of two or more groups respectively. The Pearson correlation coefficient was used to assess correlations. *p*-values generated by the correlations were adjusted for multiple testing with the false discovery rate (FDR). Differences between groups were considered significant at *p*-values < 0.05 (* *p* < 0.05, ** *p* ≤ 0.01, *** *p* ≤ 0.001). Data were analyzed using Prism version 6.0 for Windows (GraphPad Software, Inc., San Diego, CA, USA).

## 3. Results

### 3.1. TLR7 mRNA Is Expressed in Human Aortic Valves and Correlates with M2 Macrophage Markers

**Figure 1:**
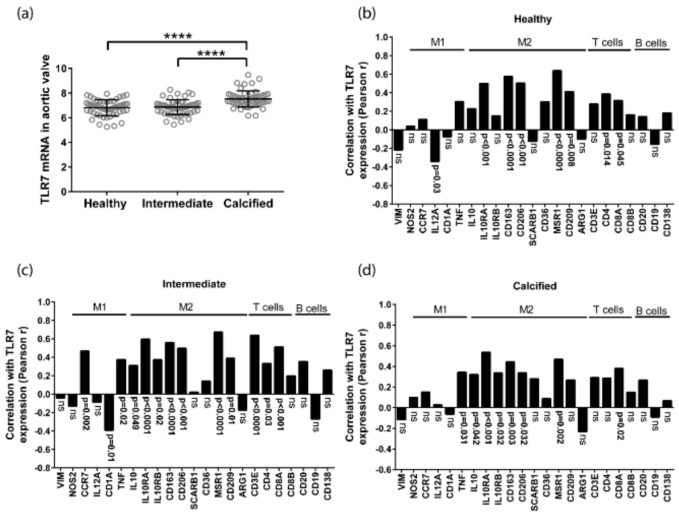
TLR7 correlates with M2 macrophage markers in healthy, intermediate, and calcified parts of the aortic valve. (**a**) TLR7 mRNA levels were significantly increased in calcified compared with healthy and intermediate parts of the aortic valve (*n* = 55). 1-way RM ANOVA with Holm-Sidak post-hoc test, **** *p* ≤ 0.0001. (**b**–**d**) Correlation analysis of TLR7 mRNA levels with mRNA levels of the VIC marker gene vimentin as well as immune cell markers in healthy (**b**), intermediate (**c**), and calcified (**d**) parts of human aortic valves (*n* = 55). Pearson correlation and false discovery rate (FDR) adjusted *p*-values are presented.

TLR7 mRNA levels were significantly higher in the calcified part of the valve compared with the healthy and intermediated parts (Figure 1a). There was a 1.77 (CI 1.57-1.97) and 1.80 (CI 1.53-2.07) fold increase of TLR7 in the calcified compared to the healthy and intermediate parts, respectively (Table 1).

TLR7 mRNA levels were significantly and positively correlated with the CD163, MRC1, MSR1, and CD209 mRNA levels, all of which are genes expressed by the anti-inflammatory macrophage subset M2 (Figure 1b,c). This correlation was found in all the different parts of the valves. There was no association of TLR7 with vimentin mRNA levels, a gene expressed by VICs (Figure 1b–d). Vimentin is a type III intermediate filament (IF) protein expressed in several cell types including VICs [25]. In addition, TLR7 mRNA levels were correlated with the T cell markers CD3 and/or CD4, CD8 in the different parts of the valves (Figure 1b,c). The analysis showed significant correlation between the T cell markers in the dissected part classified as intermediate, regarding calcification state. No association was observed between the B cell markers CD20 and CD19 with TLR7 in any part of the valves. Similarly, there was no significant association of TLR7 mRNA levels with the plasma cell marker CD138 (Figure 1b,c).

### 3.2. TLR7 Co-Localizes with T Cells and the M2 Macrophage Subset in Aortic Valves

**Figure 2:**
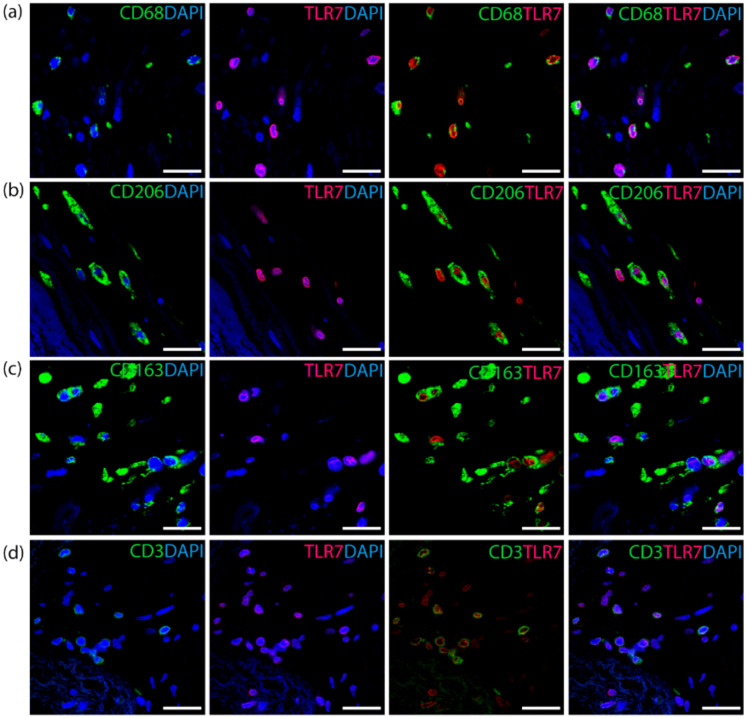
Co-localization of TLR7 and macrophage markers in human aortic valves, visualized by immunofluorescence staining. (**a**) TLR7 (red) is expressed in CD68^+^ (green) cells. (**b**) double staining of TLR7 (red) and CD206 (green). (**c**) co-localization of TLR7 (red) and CD163 (green). (**d**) TLR7 (red) is expressed in CD3^+^ (green) T cells. Nuclei are stained blue. Scale bars: 20 µm.

Double immunofluorescence analysis showed co-expression of TLR7 with T cells and M2 macrophage markers. TLR7 co-localized with the pan-macrophage marker CD68 (Figure 2a) and the M2 macrophage scavenger receptors CD206 (Figure 2b) and CD163 (Figure 2c), which were both highly correlated with TLR7 on transcript level (Figure 1b–d). Furthermore, TLR7 was co-localized with the T-cell marker CD3 (Figure 2d).

### 3.3. The TLR7 Agonist Imiquimod Alters Ex Vivo Cytokine Secretion from Human Aortic Valves

**Figure 3:**
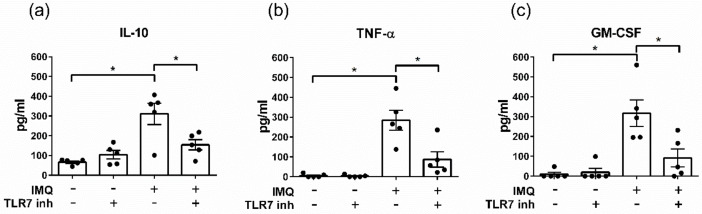
Ex vivo stimulation of human aortic valve tissue with the TLR7 agonist IMQ alters cytokine secretion. Human stenotic aortic valve tissue was stimulated ex vivo with the TLR7 ligand imiquimod (IMQ; 12.5 µg/mL). Levels of (**a**) IL-10, (**b**) TNF-α, and (**c**) GMCSF were significantly increased in the culture medium after 20 h. The effects of IMQ were significantly attenuated by the TLR7 antagonist ODN 20958 (5 µM). Data are presented as mean ± SEM. 1-way RM ANOVA with Holm-Sidak post-hoc test, * *p* < 0.05.

To investigate the effect of TLR7 activation in CAVS, an ex vivo stimulation protocol was used. Freshly removed stenotic aortic valves were chopped into small pieces and stimulated with the TLR7 agonist IMQ (12.5 µg/mL). The tissue in each well represented a mixture of healthy, intermediate, and calcified parts. TLR7 stimulation increased the secretion of IL-10, TNF-α, and GMCSF in the culture medium (Figure 3a–c). The effect of IMQ was inhibited by the TLR7 antagonist ODN 20958 for all measured cytokines (Figure 3a–c).

### 3.4. Secretion of IL-10 and TNF-a from Macrophages 

**Figure 4:**
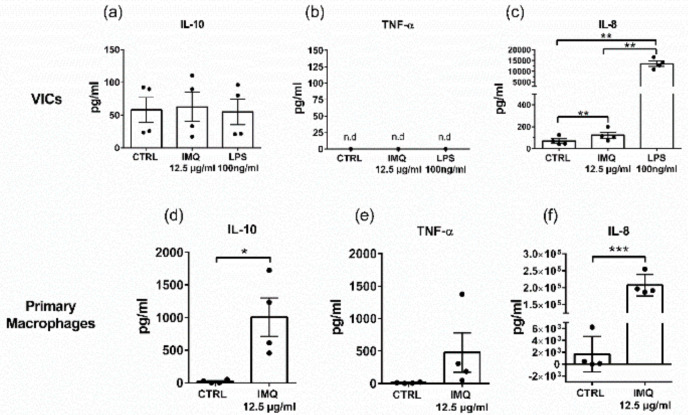
Cytokine response from valvular interstitial cells (VICs) and primary macrophages upon in vitro stimulation with TLR7 ligand. VICs were isolated from four aortic valves derived from patients with aortic valve stenosis. Primary human macrophages were derived from monocytes isolated from blood from four healthy donors. IL-10, TNF-α, and IL-8 were measured in the culture medium after 20-h stimulation of VICs and primary macrophages, respectively, with 12.5 µg/mL IMQ. LPS was used as a positive control for VICs. (**a**) No significant change in IL-10 secretion was observed in VICs after IMQ stimulation. (**b**) TNF-α was below the detection level in VICs supernatant. (**c**) Significant increase of IL-8 upon stimulation with IMQ and LPS, respectively. 1-way ANOVA with Holm-Sidak post-hoc test, ** *p* < 0.01. (**d**) Increased IL-10 secretion by primary macrophages upon TLR7 activation. (**e**) No significant change in TNF-α secretion was observed in primary macrophages after IMQ stimulation. (**f**) Significant increase of IL-8 upon stimulation of primary macrophages with IMQ. Student´s paired t-test, * *p* < 0.05, *** *p* < 0.001. Data presented as mean ± SEM.

To investigate which cells within the aortic valve tissue respond to TLR7 stimulation, primary cultures of isolated human macrophages or VICs were incubated with the TLR7 ligand. The treatment significantly increased the release of IL-10 in primary macrophages (Figure 4d), while no response was detected in VICs (Figure 4a). TNF-α was not significantly increased upon IMQ stimulation of macrophages (Figure 4e), and TNF-α levels were below the detection level in VICs supernatant (Figure 4b). To show that VICs can generally respond to stimulation in our in vitro set-up, we also measured IL-8, which is known to be secreted by activated VICs and can therefore serve as positive control [26]. Upon LPS stimulation, there was a 200-fold increase of IL-8 in the supernatant of VICs, while stimulation with IMQ led to a less than 2-fold increase in secretion compared to unstimulated cells (Figure 4c).

## 4. Discussion

Innate immunity is central in both propagation and resolution of inflammation in cardiovascular disease. Here we identify the pattern recognition receptor TLR7 as part of the network of inflammatory mediators in calcific aortic valve stenosis. Using gene expression analysis as well as immunofluorescence staining, we detect TLR7 expression in explanted human aortic valves. TLR7 mRNA correlated strongly with M2 macrophage markers, which we subsequently confirmed by immunofluorescence co-staining of TLR7 with CD163 and CD206, respectively, in aortic valve tissue. There was also correlation on mRNA level between TLR7 and T-cell markers, but this correlation was not found throughout all parts of the tissue but mainly in the intermediate part. Furthermore, ex vivo stimulation of aortic valve tissue with the TLR7 ligand imiquimod led to the secretion of the anti-inflammatory cytokine IL-10 as well as TNF-α and GMCSF. In vitro stimulation of isolated primary cells indicated that the source of this cytokine response was most likely the macrophage, while VICs did not respond to imiquimod with secretion of these cytokines. Taken together, these results indicate a link between TLR7 and M2 macrophage-related clearance pathways in human aortic valves.

The role of TLR7 has previously been investigated in atherosclerosis [19,21]. Plaques from patients operated for carotid stenosis showed infiltration of TLR7+ macrophages and T cells. In the human carotid plaque, TLR7 was connected to M2 macrophages on mRNA level [20]. Furthermore, patients with a higher level of TLR7 in the plaque had better outcome [19]. In an experimental atherosclerosis model, treatment with TLR7 ligand could ameliorate atherosclerosis [21]. Although atherosclerosis and aortic valve stenosis share some risk factors and pathophysiological mechanisms they present several differences as well. Thus, it was important to decipher the role of TLR7 as part of innate immune response in this common disease. Further investigations are needed to explore whether the use of TLR7 ligand could promote M2 macrophage function and be used as treatment for CAVS.

Previous studies on the role of TLRs in human valves have mainly focused on the expression and activation of cell surface TLR receptors in VICs and connected TLR2 and 4 signaling with detrimental effect for CAVS [17,27,28]. TLR4 is the most highly expressed TLR in VICs, [28], while TLR7, the focus of the present study, is among the lowest expressed TLRs [29].

Macrophages are characterized by a high heterogeneity; the two main subtypes are denoted M1 and M2 [30]. Traditionally, M1 macrophages are regarded as being pro-inflammatory while M2 are regarded to rather initiate tissue repair and healing [14]. Macrophage populations have important functions in cardiovascular diseases [31,32]. We show in our study an association of TLR7 expression levels with markers of the M2 macrophage subtype in aortic valves. This finding was confirmed by double immunofluorescence staining of TLR7 with two different macrophage markers, CD163 and CD206. The role of the different macrophage subsets in CAVS and specifically the mechanism by which they may affect VIC calcification is unknown. Recent in vitro data have connected the M1 macrophage subtype with the induction of a pro-osteogenic program in VICs [33]. In contrast, M2 subtype CD206^+^ macrophages regulated fibroblast activation and substantially contributed to tissue repair in a myocardial infarction (MI) mouse model [34]. In addition to their role in tissue repair, M2 macrophages also have important properties to facilitate clearance of deposited lipids through phagocytosis and apoptotic cells through efferocytosis. In a recent mouse model TLR7 ligation was shown to increase clearance of apoptotic cells after stimulation of Ly6C^+^ monocytes with a TLR7 ligand in C57BL6 mice [35]. In addition, an in vitro study indicated that TLR7 ligands shift macrophages toward an M2-like long-lived macrophage subtype cell [36]. Taken together, these studies support our finding of TLR7 expression in macrophages with clearing properties. 

The role of the different T-cell subsets has been previously investigated in CAVS. CD4^+^ and CD8^+^ T cells have been identified in advanced calcified aortic valve. Increased numbers of CD8^+^ T cells were localized in calcification areas in human calcific valves. In the same study, it was shown that secretion of IFN-γ inhibited osteoclast function and hence promoted the progression of CAVS [37]. However, CD8^+^ T cells have been associated with protective functions in atherosclerosis [38]. Furthermore, regulatory T cells were increased in the circulation of patients with severe calcific aortic disease, possibly as a compensatory mechanism to suppress extensive inflammatory responses [39,40]. In our study, we show expression of TLR7 in T cells, but more studies are required to decipher the functions of TLR7 in T cells related to CAVS.

Incubation of human aortic valve tissue with a TLR7 ligand in an ex vivo assay led to increased secretion of IL-10, TNF-α, and GMCSF to the conditioned medium. These three cytokines have previously been identified in a similar ex vivo stimulation assay of human carotid plaques after screening for 20 cytokines and showed them to increase in a dose-dependent manner [19]. IL-10 is an important immunomodulatory cytokine that has been suggested to ameliorate cardiovascular diseases such as atherosclerosis [41,42]. IL-10 inhibits the production of pro-inflammatory cytokines such as IFN-γ and IL-6 [43] and is actively involved in restoring tissue homeostasis. Furthermore, it has been shown that phagocytes secrete IL-10 upon efferocytosis [44].

We also observed an increased secretion of TNF-α upon ex vivo stimulation of aortic valve tissue with TLR7 ligand. TNF-α has been connected to pro-osteogenic pathways in in vitro cultures of VICs [45]; the role of TNF-α in cardiovascular disease is, however, contradictory [46]. Overexpression of TNF-α in a mouse model of genetic heart failure leads to cardioprotection through the induction of genes related to the intermediate filament cytoskeleton [47]. Controlled TNF-α expression can convey tissue repair and cardioprotection; however, sustained expression is connected to detrimental outcome in cardiovascular diseases [47].

To gain more insight into the type of cell that could possibly respond to TLR7 ligation in stenotic human aortic valves, we investigated two cell types that are present in this tissue; VICs, the major structural cells in aortic valves, and macrophages, which infiltrate the tissue during pathogenesis of CAVS and were abundantly detected in this study using immunofluorescence staining of aortic valve tissue. When we stimulated isolated VICs in vitro via TLR7, we did not observe a significant increase in IL-10 or TNF-α release. Furthermore, we did not observe any correlation on the mRNA level between TLR7 and the VICs marker vimentin in our microarray analysis of CAVS. On the other hand, when we stimulated primary macrophages isolated from the blood of healthy donors with the TLR7 ligand, we saw a significant increase in the secretion of IL-10, TNF-α, and GMCSF. These data further strengthen the possibility that effects mediated via TLR7 signaling in CAVS are attributed to macrophages rather than VICs. However, we cannot exclude that other infiltrating immune cells such as T or B lymphocytes also contribute to the TLR7 ligand response that we report in stenotic aortic valve tissue. In contrast, VICs secreted almost 200-fold increased levels of IL-8 after stimulation with the TLR4 ligand LPS, while TLR7 ligation resulted only in a modest, less than 2-fold increase in IL-8 secretion. These data indicate that TLR4 plays a more important role than TLR7 in VICs. Indeed, a recent study has confirmed the lack of TLR7 response in VICs by measuring NF-κB activation [48]. Our immunofluorescence analysis showed expression of TLR7 in T cells. Stimulation of blood-derived CD4^+^ and CD8^+^ T cells with IMQ has previously been shown not to significantly alter either IL-10 or TNF-α secretion [19]. A possible explanation is that the tissue microenvironment affects the activation stage of the T cells and thus responsiveness to TLR7 ligand.

## 5. Conclusions

In summary, TLR7 is upregulated during CAVS pathogenesis in human aortic valves and associated with the M2 macrophage subtype. Ex vivo aortic valve tissue challenged with TLR7 ligand led to the increased production of cytokines including the immunomodulatory cytokine IL-10. These results connect TLR7 activation in CAVS to immunomodulatory and anti-inflammatory pathways.

## Figures and Tables

**Table 1:** Gene expression of TLR7 in healthy, intermediate, and calcified parts of the aortic valve.

		Calcified vs Healthy	Calcified vs Intermediate	Intermediate vs Healthy
TLR7	Fold change	1.77	1.80	1.05
	CI	1.57–1.97	1.53–2.07	0.93–1.17
